# Exploring the Role of Posttranslational Modifications in Spinal and Bulbar Muscular Atrophy

**DOI:** 10.3389/fnmol.2022.931301

**Published:** 2022-06-03

**Authors:** Neha Gogia, Luhan Ni, Victor Olmos, Fatema Haidery, Kimberly Luttik, Janghoo Lim

**Affiliations:** ^1^Department of Genetics, Yale School of Medicine, Yale University, New Haven, CT, United States; ^2^Yale College, Yale University, New Haven, CT, United States; ^3^Department of Neuroscience, Yale School of Medicine, Yale University, New Haven, CT, United States; ^4^Interdepartmental Neuroscience Program, Yale University, New Haven, CT, United States; ^5^Program in Cellular Neuroscience, Neurodegeneration and Repair, Yale School of Medicine, Yale University, New Haven, CT, United States; ^6^Yale Stem Cell Center, Yale School of Medicine, Yale University, New Haven, CT, United States

**Keywords:** spinal and bulbar muscular atrophy (SBMA), androgen receptor (AR), posttranslational modification (PTM), neurodegeneration, polyglutamine disease

## Abstract

Spinal and Bulbar Muscular Atrophy (SBMA) is an X-linked adult-onset progressive neuromuscular disease that affects the spinal and bulbar motor neurons and skeletal muscles. SBMA is caused by expansion of polymorphic CAG trinucleotide repeats in the *Androgen Receptor* (*AR*) gene, resulting in expanded glutamine tract in the AR protein. Polyglutamine (polyQ) expansion renders the mutant AR protein toxic, resulting in the formation of mutant protein aggregates and cell death. This classifies SBMA as one of the nine known polyQ diseases. Like other polyQ disorders, the expansion of the polyQ tract in the AR protein is the main genetic cause of the disease; however, multiple other mechanisms besides the polyQ tract expansion also contribute to the SBMA disease pathophysiology. Posttranslational modifications (PTMs), including phosphorylation, acetylation, methylation, ubiquitination, and SUMOylation are a category of mechanisms by which the functionality of AR has been found to be significantly modulated and can alter the neurotoxicity of SBMA. This review summarizes the different PTMs and their effects in regulating the AR function and discusses their pathogenic or protective roles in context of SBMA. This review also includes the therapeutic approaches that target the PTMs of AR in an effort to reduce the mutant AR-mediated toxicity in SBMA.

## Introduction

Insights obtained from the human genome sequencing has made it evident that numerous and complex biological functions required for life cannot be answered only by a limited number of protein coding genes. In order for a cell to perform its numerous required functions, complex molecular mechanisms, including alternative splicing and posttranslational modifications (PTMs), allow the genome to drastically increase its ability to produce proteins that can meet the demand of life. Both these mechanisms allow the cell to produce multiple different functional proteins from a single gene. Alternative splicing accomplishes this through modification of pre-mRNA constructs, producing differently spliced mRNAs that are then translated to different mature proteins. In contrast, PTMs are chemical modifications that are added to the mature proteins to change or modify their function. PTMs involve addition of acetyl, methyl, phosphate groups, or ubiquitin molecules *via* covalent or enzymatic addition to the targeted amino acids. These forms of modifications are required to regulate proteome homeostasis, biological pathways, or other cellular functions (Karve and Cheema, 2011). Dysregulated or altered PTMs have been implicated in diverse neurodegenerative diseases (Chen and Feany, 2005; Arakhamia et al., 2020; Gupta et al., 2021; Sternburg et al., 2022), including polyglutamine (polyQ) diseases (Lieberman et al., 2002; Mookerjee et al., 2009; Pennuto et al., 2009; Ju et al., 2014; Martin et al., 2018; Ratovitski et al., 2021). PolyQ diseases are a family of neurodegenerative disorders that are caused by CAG trinucleotide repeat expansion in their respective disease-associated genes (La Spada et al., 1991; Macdonald et al., 1993; Orr et al., 1993; Koide et al., 1994; Lorenzetti et al., 1997; Nakamura et al., 2001; Lieberman et al., 2019). This group encompasses nine diseases, including Huntington’s Disease (HD), Dentatorubral-pallidoluysian atrophy (DRPLA), spinocerebellar ataxias (SCA 1, 2, 3, 6, 7, and 17), and spinal and bulbar muscular atrophy (SBMA) (Orr and Zoghbi, 2007; Lieberman et al., 2019).

**FIGURE 1:**
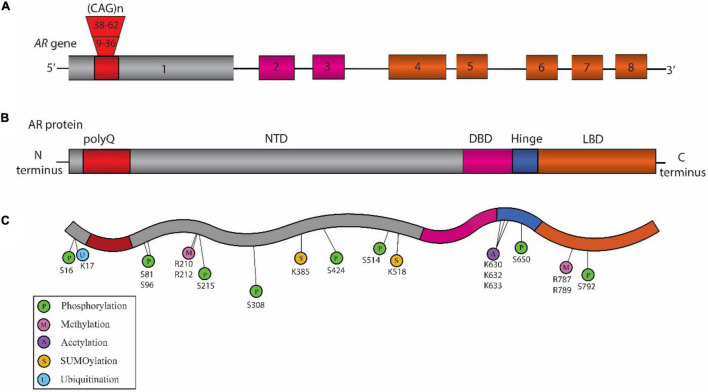
Schematic representation of the structure of *Androgen Receptor* (*AR*) gene, protein, and posttranslational modifications (PTM) sites associated with Spinal and Bulbar Muscular Atrophy (SBMA) pathogenesis. **(A)** Structure of *AR* gene: The *AR* gene is located on the X chromosome and is comprised of eight exons. The color-coded exons indicate the major functional domains in the translated AR protein. **(B)** Domain architecture of AR: Schematic illustration showing the key domains that include the N-terminal domain (NTD, shown in gray), the DNA binding domain (DBD, pink), a short hinge region (blue), and the C-terminal ligand binding domain (LBD, orange) in the AR protein. **(C)** SBMA-associated PTM sites on the AR: The sites include phosphorylation sites (P, green), methylation sites (M, pink), acetylation sites (A, purple) SUMOylation sites (S, yellow), and ubiquitination sites (U, blue). The position of these PTM sites is indicated below their respective circles.

Spinal and bulbar muscular atrophy, also known as Kennedy’s disease, is an X-linked adult-onset progressive neuromuscular disorder (Kennedy et al., 1968; La Spada et al., 1991) with an estimated prevalence of 1:300,000 (La Spada, 1993). It is a monogenic disorder caused by CAG repeat expansion in the first exon of the *Androgen Receptor* (*AR*) gene (La Spada et al., 1991) (Figure 1). The location of *AR* gene on the X chromosome as well as the presence of androgenic hormones makes SBMA more prevalent in the males; however, homozygous females can exhibit symptoms which are milder than those observed in the males (Katsuno et al., 2012). The healthy individuals normally carry 9 to 36 CAG repeats, while the individuals affected with SBMA have 38 to 62 CAG repeats in *AR*. The disease symptoms occur around 30 to 50 years of age (Kennedy et al., 1968; Atsuta et al., 2006) with a strong correlation between the age of disease onset and CAG repeat length (Igarashi et al., 1992; Atsuta et al., 2006). The disease pathology includes behavioral tremors, muscle cramps, increased levels of creatine kinase, motor neuron loss, and skeletal muscle degeneration (Kennedy et al., 1968; Soraru et al., 2008).

Since the genetic cause of SBMA was identified, several *in vivo* and *in vitro* models have been developed that recapitulate many aspects of SBMA phenotypes (Pennuto and Basso, 2016). Conservation of the basic genetic machinery across the species as well as anatomical and physiological similarities allow these models to provide valuable insights into the disease pathology and pathogenesis. For *in vivo* animal models, the first transgenic mouse model used to study SBMA contained 45 CAG repeats in the *AR*; however, it did not show the SBMA-related phenotype due to the low transgene expression (Bingham et al., 1995). Another transgenic mouse model developed, which carried 45 CAG repeats in *AR*, showed repeat instability (La Spada et al., 1998). Subsequently, additional mouse models with varying repeat lengths were developed that recapitulated the diverse aspects of SBMA pathology and opened the door toward the mechanistic understanding of the disease and the development of therapeutics to treat SBMA (Abel et al., 2001; Adachi et al., 2001; Katsuno et al., 2002; McManamny et al., 2002; Chevalier-Larsen et al., 2004; Sopher et al., 2004; Yu et al., 2006; Monks et al., 2007; Cortes et al., 2014a; Chua et al., 2015; Ramzan et al., 2015; Zboray et al., 2015; Badders et al., 2018). In addition to the mouse models, invertebrate *Drosophila melanogaster* (fruit fly) models have also been developed and used to study the SBMA pathology (Chan et al., 2002; Takeyama et al., 2002; Matsumoto et al., 2005; Funderburk et al., 2009; Suzuki et al., 2009; Palazzolo et al., 2010; Jochum et al., 2012; Scaramuzzino et al., 2015; Todd et al., 2015; Bott et al., 2016; Badders et al., 2018; Nath et al., 2018). Furthermore, in addition to the animal models used for *in vivo* studies, *in vitro* approaches have also proven to be beneficial in gaining deeper insights into the mechanisms contributing to the SBMA pathology. Various cell lines, either transfected with expanded polyQ-AR or human patients-derived, have widely been used in this aspect (Brooks et al., 1997; Simeoni et al., 2000; Walcott and Merry, 2002; Malena et al., 2013; Cortes et al., 2014b; Dossena et al., 2014; Grunseich et al., 2014; Scaramuzzino et al., 2015; Milioto et al., 2017). A number of the primary findings with a significant contribution toward unraveling the role of PTMs in regulating the AR function and SBMA pathogenesis using diverse model systems are discussed in this review.

## Androgen Receptor and SBMA

The *AR* gene is located on the X chromosome (Xq11.2-q12) in humans (Brown et al., 1989). Its sequence comprises a total of eight exons that encode for the AR protein (Figure 1). AR is a nuclear transcription factor that belongs to the steroid hormone receptor family (Tsai and O’Malley, 1994). Structurally, AR is broadly divided into 3 distinct regions (Figure 1). The amino (N)-terminal domain (NTD) is known to regulate the transcriptional activation and regulation of AR (Jenster et al., 1995). The DNA binding domain (DBD) recognizes the specific DNA sequences, facilitates AR-DNA binding, and also controls AR dimerization (Shaffer et al., 2004). A flexible hinge region connects the DBD to the ligand binding domain (LBD). The LBD controls AR dimerization for transcriptional activation in a ligand-dependent manner (El Kharraz et al., 2021). The nuclear localization signal (NLS) is located between the DBD and the hinge region, and facilitates the translocation of AR into the nucleus (Zhou et al., 1994). The nuclear export signal (NES) located within the LBD promotes the export of AR from the nucleus to the cytoplasm (Saporita et al., 2003).

In the absence of ligands such as testosterone or dihydrotestosterone (DHT), AR exists as a complex with the heat-shock proteins (HSPs), such as HSP90 and HSP70, within the cytoplasm. However, when the ligand is present, AR protein binds the ligand, resulting in a conformational change in the AR structure that promotes dissociation from HSPs and allows it to translocate into the nucleus (Kuil et al., 1995). Within the nucleus, AR forms a dimer and binds androgen responsive elements (AREs) of the promotor regions of specific target genes and promotes their transcription (Shang et al., 2002). AR plays a key role in androgen-dependent development and maintenance of male sexual physiology (Chang et al., 2013). It is also known to play a role in diverse cellular functions, such as promoting axon remyelination in the central nervous system (Bielecki et al., 2016), motor neuron survival and degeneration (Cary and La Spada, 2008), muscle development (Wyce et al., 2010), and immune system function (Lai et al., 2012). In the context of disease, dysfunction or dysregulation of AR is known to be associated in causing multiple diseases (Matsumoto et al., 2013), including androgen insensitivity syndrome (Bevan et al., 1997), prostate cancer (Huang and Tindall, 2002), and SBMA (Suzuki et al., 2008; Beitel et al., 2013). There are multiple mechanisms relating to AR that are known to contribute to SBMA pathogenesis (Beitel et al., 2013; Cortes and La Spada, 2018). They include expansion of polyQ tract in the AR (La Spada et al., 1991), AR aggregation (Merry et al., 1998; Stenoien et al., 1999), transcriptional dysregulation (Mhatre et al., 1993; Lieberman et al., 2002), formation of polyQ-AR oligomers (Li et al., 2007), structural changes (Davies et al., 2008), interdomain interactions (Orr et al., 2010), nuclear translocation of polyQ-AR (Nedelsky et al., 2010), polyQ mediated altered AR interaction with other proteins (Pluciennik et al., 2021), and altered PTMs (Table 1). A number of these SBMA-associated pathogenic mechanisms have been implicated to be regulated by the PTMs. This review provides a thorough overview of the role of PTMs on AR in SBMA pathogenesis.

**TABLE 1:** Posttranslational modifications (PTM) sites within the Androgen Receptor (AR) that can modulate the spinal and bulbar muscular atrophy (SBMA) pathogenesis.

PTMs	Sites	Caused by	Nature	Model system	Mechanism of action	References
Phosphorylation	S16	Mutation in the FxxLF motif (F23A)	At amino (N) terminal region	Cell culture, mouse	Phosphorylation enhanced by mutation in FxxLF motif (F23A) of AR, prevented N/C interaction, and reduced the disease toxicity.	Zboray et al., 2015
	S81 and S308	Genetic mutations on AR, F23A, L26A/F27A	Point mutation	Cell culture	Phosphorylation at S81 and S308 was reported to be dependent upon N/C interaction.	(Orr et al., 2010
	S81	Nemo-Like Kinase (NLK)	Serine/threonine kinase	Cell culture, *Drosophila*	NLK phosphorylated polyglutamine (polyQ) -AR, increasing polyQ-AR aggregation and toxicity.	Todd et al., 2015
	S96	CDK2	Cyclin dependent kinase	Cell culture, mouse	CDK2 increased the phosphorylation level of polyQ-AR and disease toxicity.	Polanco et al., 2016
	S215 and S792	Phosphomimetic mutation; S215D, S792D	Point mutation	Cell culture	Phosphomimetic mutation blocked ligand dependent nuclear translocation and transcriptional activation, and reduced polyQ-AR mediated toxicity.	Palazzolo et al., 2007
	S215 and S792	Insulin-like Growth Factor 1 (IGF-1)	Growth hormone	Cell culture, mouse	IGF-1 decreased the aggregation of AR and promoted AR clearance *via* the ubiquitin-proteasome system through phosphorylation of AR by Akt.	Palazzolo et al., 2009
	S424 and S514	Double mutation at S424 and S514	Point mutation	*Drosophila*	Mutation preventing phosphorylation in polyQ-AR reduced the disease toxicity.	Funderburk et al., 2009
	S514	Mutation, S514A	Point mutation	Cell culture	Mutation prevented the phosphorylation at S514 suppressed polyQ-AR mediated cell death.	LaFevre-Bernt and Ellerby, 2003
	S650	Mutation, S650A	Point mutation	Cell culture	Reduced phosphorylation at S650 impaired the nuclear export of polyQ-AR.	Arnold et al., 2019
Methylation	R210, R212, R787, R789	Protein Arginine Methyl Transferase 6 (PRMT6)	A methyl transferase	Cell culture, *Drosophila*	PRMT6 led to arginine methylation of AR with enhancement of AR transactivation, resulting in disease toxicity.	Scaramuzzino et al., 2015
Acetylation	K 630/632/633	Sirtuin-1 (SIRT1)	NAD + dependent histone deacetylase, a nuclear protein	Cell culture	SIRT1 deacetylated AR at lysine 630/632/633 and provided protection against polyQ-AR mediated toxicity.	Montie et al., 2011
	K630/632, K633	Mutation of K630/632 and K633 to alanine	Point mutation	Cell culture	Acetylation deficient mutation delayed the ligand dependent nuclear translocation and promoted AR aggregation.	Thomas et al., 2004
Ubiquitination	K17	Ubiquitin-specific protease7 (Usp7)	Deubiquitinase	Cell culture, *Drosophila*, mouse	Downregulation of Usp7 lowered AR aggregation-mediated toxicity and ameliorated SBMA phenotypes.	Pluciennik et al., 2021
SUMOylation	–	Uba2	SUMO-1 activating enzyme	*Drosophila*	Overexpression of Uba2.C175S resulted in increase of polyQ-AR induced toxicity.	Chan et al., 2002
	K385 and K518	Small Ubiquitin-like Modifier (SUMO)	Protein modifier	Cell culture	SUMOylation reduced polyQ-AR aggregation.	Mukherjee et al., 2009
	K385 and K518	Mutation of lysine sites K385 and K518 to arginine	Point mutation	Cell culture, mouse	Disruption of polyQ-AR SUMOylation enhanced transcriptional function of ligand induced polyQ-AR and rescued the disease pathology.	Chua et al., 2015

## Posttranslational Modifications

Posttranslational modifications are biochemical modifications that add or remove specific chemical groups, such as acetyl, methyl, phosphate, or ubiquitin, to specific proteins that change the properties of those target proteins. PTMs help expand the potential of a protein to carry out cellular functions beyond its functional capacity (Ramazi and Zahiri, 2021). Aberrant PTMs of specific proteins or dysregulation of PTM homeostasis have been implicated in modulating pathophysiology of polyQ diseases, such as SCA1 (Ju et al., 2013; Ju et al., 2014; Kang et al., 2015), HD (Martin et al., 2018), DRPLA (Yazawa, 2000), and SBMA (Anbalagan et al., 2012) (Table 1) among others. The AR activity can be modulated by multiple PTMs, including phosphorylation, acetylation, methylation, ubiquitination, and SUMOylation, and pharmacological modulation of AR activity through PTMs has been observed to mitigate the aspects of polyQ-AR mediated disease toxicity in SBMA (Tables 1, 2).

**TABLE 2:** Therapeutics targeting PTM as treatment strategies for SBMA.

PTM	Therapeutics	Description	Model system	Mechanism of action	References
Phosphorylation	PACAP analog	Activator of AC/PKA signaling pathway	Cell culture, mouse	Activation of AC/PKA negatively regulated CDK2 mediated phosphorylation of polyQ-AR and reduced polyQ-AR aggregation and toxicity.	Polanco et al., 2016
	Forskolin		Cell culture		
Phosphorylation	IGF-1	Insulin-Like Growth Factor 1	Cell culture	IGF-1 enhanced clearance of polyQ-AR aggregates *via* ubiquitin-proteasome system in a mechanism mediated by phosphorylation of AR by Akt.	Palazzolo et al., 2009
	mIGF-1	A muscle specific isoform of IGF-1	Mouse	Expression of mIGF-1 decreased AR aggregation and ameliorated SBMA phenotypes.	
	Mecasermin rinfabate	Recombinant human IGF-1 and IGF-1 binding protein 3	Mouse	Mecasermin rinfabate attenuated the mutant AR mediated disease toxicity *via* activation of Akt.	Rinaldi et al., 2012
	BVS857, Drug	IGF1-memetic	Human	- The protective function of IGF-1 against mutant AR was observed to be dependent upon phosphorylation of AR by Akt. - No significant differences were found in this study in terms of muscle strength and muscle function.	Grunseich et al., 2018

### Phosphorylation

Phosphorylation and dephosphorylation are the most common reversible chemical modifications that are carried out broadly by kinases and protein phosphatases, respectively (Cohen, 2002). They play crucial roles in regulating protein function, stability, localization, and interaction, etc. Phosphorylation within the AR primarily occurs at amino acids residues serine (S), threonine (T), and tyrosine (Y) (Gioeli and Paschal, 2012; Ardito et al., 2017). There are several AR phosphorylation sites which have been shown to modulate AR activity and thereby SBMA pathogenesis as evidenced from different *in vivo* and *in vitro* models (Figure 1; Table 1). The key phosphorylation sites within the AR protein that have been implicated in SBMA pathogenesis are discussed below.

#### S16

Androgen receptor amino (N)- and carboxyl (C)-terminal interdomain (N/C) interaction is known as one of the potential mechanisms that contributes to SBMA pathogenesis (Orr et al., 2010). The potential role of phosphorylation at S16 site in SBMA has been studied in this aspect (Zboray et al., 2015). Phosphorylation of AR at S16 was found to be enhanced when the N-terminal FxxLF motif of polyQ-AR was mutated (F23A) (Zboray et al., 2015). This mutation disrupted the interaction between the N-terminal FxxLF motif and C-terminal Activation Function-2 (AF-2) domain and ameliorated the behavioral and pathological phenotypes as seen in transgenic mouse models of SBMA (Zboray et al., 2015). In addition to that, prevention of phosphorylation at S16 site by S16A mutation eliminated the protective effects by F23A mutation in PC12 cells (Zboray et al., 2015). Taken together, these findings suggest that the protective effect of AR F23A is dependent on S16 phosphorylation (Zboray et al., 2015).

#### S81 and S308

Similarly, the role of phosphorylation at S81 and S308 sites has also been investigated in AR N/C interaction and AR aggregation in SBMA. In contrast to S16, phosphorylation at S81 and S308 was reported as a positive marker for the N/C interaction of polyQ-AR (Orr et al., 2010). In a study conducted to investigate whether the N/C interaction was required for polyQ-AR mediated disease toxicity and polyQ-AR aggregation, Orr et al. (2010) utilized both bicalutamide that is a transcriptional antagonist of AR and genetic mutations (F23A, L26A/F27A) that can inhibit the DHT induced interaction between N/C. Treatment with bicalutamide was found to disrupt the AR N/C interaction and prevent phosphorylation of S81 and S308 in the presence of DHT (Orr et al., 2010). It was also found to reduce the DHT-induced disease toxicity and aggregation of AR in cell culture models of SBMA (Orr et al., 2010). Similarly, the genetic mutations (F23A, L26A/F27A) inhibiting the N/C interaction were shown to prevent the AR phosphorylation at S81 and S308 *in vitro* (Orr et al., 2010). Overall, this study indicated that AR phosphorylation at S81 and S308 is dependent upon the AR N/C interaction (Orr et al., 2010).

A subsequent study found that AR S81 phosphorylation contributes to SBMA phenotypes (Todd et al., 2015). Nemo-like kinase (NLK) was found to regulate the phosphorylation levels of the mutant AR at S81 and S308 (Todd et al., 2015). Specifically, overexpression of NLK increased, while NLK reduction decreased, the S81 phosphorylation and polyQ-AR aggregates formation. Furthermore, the phosphorylation-resistant mutation AR-S81A was found to ameliorate the effects of NLK on AR aggregation *in vitro*, thereby indicating that NLK promotes mutant AR aggregation in part through S81 phosphorylation (Todd et al., 2015). To check the effect of NLK mediated change in S81 phosphorylation on SBMA phenotypes *in vivo*, the study utilized a *Drosophila* model of SBMA expressing a truncated N-terminal fragment of AR (trAR112Q) (Todd et al., 2015). Loss of one copy of *Drosophila* homolog of *NLK* (*nmo* in flies) reversed the severe retinal degeneration phenotypes in the trAR112Q SBMA fly model (Todd et al., 2015). The trAR112Q fragment possesses two potential NLK target sites (S81 and S94/96) and the mutation S94/96A did not influence the NLK-mediated effect on AR aggregation in cells (Todd et al., 2015). Taken together, this study suggested that AR phosphorylation at S81 mediated by NLK may play an important role in SBMA pathology (Todd et al., 2015).

#### S96 (S94)

The role of phosphorylation at S96 has been shown in regulating the polyQ-AR protein stabilization (Polanco et al., 2016). In this study, Polanco et al. (2016) reported that cyclin-dependent kinase 2 (CDK2) phosphorylates polyQ-AR at S96 site, which enhances the mutant AR stability and toxicity. They also showed that activation of adenylyl cyclase (AC)/protein kinase A (PKA) signaling increased the turnover of polyQ-AR protein and decreased the mutant AR aggregation *in vitro*, in a manner that potentially involves inhibition of CDK2 (Polanco et al., 2016). Further, to test the effect of phosphorylation on polyQ-AR protein stabilization, S96A substitution was used in the study and was found to decrease the accumulation of mutant AR protein (Polanco et al., 2016). In order to translate these findings into a possible therapy for SBMA, the group utilized an analog of pituitary adenylyl cyclase activating polypeptide (PACAP), a potent activator of AC/PKA signaling. Administration of PACAP analog was found to reduce the accumulation of S96 phosphorylated and total polyQ-AR and attenuate the phenotypes in knock-in mouse model of SBMA (Polanco et al., 2016), further indicating the importance of S96 phosphorylation site as a potential PTM therapeutic target.

#### S215 and S792

Phosphorylation at S215 and S792 has been reported to be associated with regulating the nuclear localization, transcriptional function, and toxicity of polyQ-AR (Palazzolo et al., 2007). S215 and S792 on AR belong to Akt (protein kinase B) consensus sites and have shown to be phosphorylated by Akt/PKB kinases (Lin et al., 2001). An *in vitro* study reported that the phosphomimetic substitution of S215 and S792 with aspartate reduced the ligand binding, ligand-dependent transactivation, nuclear localization, and polyQ-AR mediated toxicity (Palazzolo et al., 2007). Akt was observed to increase the phosphorylation of polyQ-AR and suppress the DHT induced dependent AR transcriptional activity *in vitro* (Palazzolo et al., 2007). Treatment of insulin-like growth factor 1 (IGF-1) was found to lower the polyQ-AR aggregation and toxicity *in vitro*, which is mediated by the phosphorylation of AR at S215 and S792 (Palazzolo et al., 2007). Along the same line, phosphorylation-resistant mutation (S215A, S792A) of polyQ-AR were observed to decrease the protective effects of IGF-1 *in vitro* (Palazzolo et al., 2007). Another study showed that overexpression of IGF-1 reduced the polyQ-AR aggregates through ubiquitin-proteasome system in Akt-mediated AR phosphorylation dependent manner *in vitro* (Palazzolo et al., 2009). The expression of muscle specific isoform of IGF-1 could increase activation of Akt and AR phosphorylation but decreased the AR aggregation, and improved the behavioral and pathological phenotypes in a mouse model of SBMA (Palazzolo et al., 2009). Taken together, these studies indicated that increased phosphorylation of AR at S215 and S792 is able to reduce polyQ-AR toxicity in SBMA models.

#### S424 and S514

Phosphorylation at S514 has been implicated in the formation of polyQ-AR fragments and polyQ-AR mediated cellular toxicity (LaFevre-Bernt and Ellerby, 2003). Findings from *in vitro* SBMA models have shown that the phosphorylation of AR at site S514 is pathogenic and increases the ability of caspase-3 to cleave AR and form toxic polyQ-AR fragments, thereby resulting in cell death *in vitro* (LaFevre-Bernt and Ellerby, 2003). Additional studies in fly models identified reduced levels of toxicity upon mutations of two phosphorylation sites in polyQ-AR at S424 and S514 (ARQ77dm) compared to ARQ77 (Funderburk et al., 2009). Interestingly, the double mutation at these sites in wild-type AR (ARQ22dm) altered the nuclear translocation, transactivation, and enhanced hormone-dependent AR aggregation *in vitro* (Funderburk et al., 2009). Similarly in fly model, overexpression of ARQ22dm resulted in severe degeneration of photoreceptor neurons and affected the survival and behavior in flies (Funderburk et al., 2009). This indicates that phosphorylation at S424 and S514 may have distinct effects on wild-type AR and polyQ-AR, respectively; thus, further investigations are needed to fully understand the role of phosphorylation at these sites within the AR in relation to SBMA.

#### S650

Phosphorylation of AR at S650 is known to regulate the nuclear export of wild-type AR (Gioeli et al., 2006; Chen et al., 2009). A study conducted *in vitro* found that polyQ-AR showed reduced phosphorylation at S650 site and an impaired nuclear export (Arnold et al., 2019). Phosphorylation-resistant mutation at S650 site (S650A) was observed to further impair the nuclear export of polyQ-AR *in vitro* (Arnold et al., 2019). However, the phosphomimic mutation (S650D) did not showed significant rescue of impaired nuclear export of polyQ-AR (Arnold et al., 2019). Thus, the precise role of S650 site in regulating the AR nuclear export and in polyQ-AR mediated toxicity in SBMA requires more investigation.

Taken together, researches conducted in the past have certainly made it clear that the phosphorylation on AR has critical roles in polyQ-AR toxicity in phosphorylation site-dependent manner. Identification and further investigation of the role of phosphorylation sites of AR in context of SBMA still require detailed studies.

### Methylation

Methylation at arginine residues has been implicated in context of polyQ disease pathogenesis (Basso and Pennuto, 2015; Scaramuzzino et al., 2015; Migazzi et al., 2021). Arginine methylation is carried out by a group of enzymes known as protein arginine methyltransferases (PRMTs) (Bedford and Clarke, 2009; Blanc and Richard, 2017). PRMTs leads to the addition of a methyl group to arginine, thereby resulting in change in the structure of arginine (Bedford and Clarke, 2009). *In vitro* studies have shown PRMT6 acts as a modifier of polyQ-AR toxicity *in vivo* and *in vitro* (Scaramuzzino et al., 2015). PRMT6 methylates the arginine residues of polyQ-AR at Akt consensus site motif RXRXXS (Table 1; Figure 1) (Scaramuzzino et al., 2015). PRMT6 serves as a cofactor of AR and its activity gets enhanced in the polyQ-AR background *in vitro* (Scaramuzzino et al., 2015). It colocalizes, forms a complex with AR, promotes transactivation of AR, increases the polyQ-AR toxicity, and contributes to SBMA disease pathology (Scaramuzzino et al., 2015). To test these findings *in vivo*, a fly model of SBMA was used in the study. Genetic knockdown of DART8 (PRMT6 ortholog in flies) was found to suppress the polyQ-AR induced neurodegeneration phenotypes in the fly eye (Scaramuzzino et al., 2015).

In addition to PRMT6, the role of other members of the PRMT family, such as PRMT1 (Koh et al., 2001; Tang et al., 2022), PRMT2 (Meyer et al., 2007), PRMT 5 (Deng et al., 2017), and PRMT 10 (Harada et al., 2015), has also been studied in regulating the AR function or expression. PRMT7 has been shown to recognize the lysine- and arginine-rich regions within the RXR motif (similar to the Akt consensus site motif RXRXXS on AR) (Feng et al., 2013); however, the precise roles of these PRMTs specifically in context of SBMA pathogenesis still need to be ascertained. Furthermore, SET9 methyltransferase has been shown to interact and regulate the AR *via* methylation at lysine residues (Gaughan et al., 2011), which indicates additional modes of AR regulation that may be therapeutically exploitable in context of SBMA. Altogether, results from these studies indicated AR methylation as a modulator of SBMA disease toxicity. Further understanding of the role of other methylation enzymes, their specific methylation sites, and their effects on SBMA pathogenesis is needed.

### Acetylation

Acetylation is a process that involves the transfer of acetyl groups by acetyl-CoA to a specific site on a polypeptide sequence (Drazic et al., 2016). Acetylation is known to act as a modulator of pathogenesis in multiple neurodegenerative diseases (Montie et al., 2011; Vinueza-Gavilanes et al., 2020; Gottlieb et al., 2021), including SBMA (Montie et al., 2011). Here, we discuss the acetylation sites K630, K632, K633 within the AR that have been studied in the context of SBMA (Figure 1; Table 1).

An *in vitro* cell culture study investigated the role of AR acetylation in SBMA pathology and highlighted the potential beneficial roles of SIRT1 and AR deacetylation on SBMA phenotypes (Montie et al., 2011). Sirtuins, initially identified as silence information regulators (SIRs), are a family of NAD + dependent class III histone deacetylases that have been implicated to play role in aging and neurodegenerative diseases (Jesko et al., 2017). Mammals possess seven Sirtuins (SIRT 1–7) (Haigis and Sinclair, 2010). Among them, SIRT1, a nuclear protein (Sun and Fang, 2016), has been shown to act as a deacetylase of AR (Fu et al., 2006). Expression of SIRT1 was observed to deacetylate polyQ-AR at K630/632/633 sites and ameliorate SBMA phenotypes *in vitro* (Montie et al., 2011). Genetic mutations preventing this acetylation of polyQ-AR at K630/632/633 residues were found to reduce the DHT induced protein stabilization of poyQ-AR and suppress the polyQ-AR aggregation and toxicity *in vitro* (Montie et al., 2011), thereby strongly suggesting acetylation as potential target for the development of effective therapeutics. Interestingly, a study conducted *in vitro* has shown that acetylation site mutations at K630, 632, and 633 residues (K630A, K632A/K633A) in the wild-type AR, resulted in delayed nuclear localization (Thomas et al., 2004). AR with K632A/K633A mutation also showed DHT-dependent misfolding and aggregation, as seen in the polyQ-AR background (Thomas et al., 2004). Furthermore, the study demonstrated that aggregates of AR with K632A/K633A mutation, colocalized with chaperons and inhibited the proteasomal activity (Thomas et al., 2004). Taken together, these findings suggested that AR acetylation affects the AR regulation and may contribute to polyQ-AR mediated toxicity. However, the precise role of AR acetylation in wild-type and polyQ-AR background remains to be elucidated and requires detailed investigation.

### Ubiquitination

Ubiquitination is a PTM carried out by ubiquitination enzymes E1, E2, and E3, involves covalent attachment of ubiquitin molecules to the target proteins, and aids in regulating biological functions within the cell (Wilkinson, 1987; Swatek and Komander, 2016). The role of ubiquitination as a PTM and ubiquitination site K17 within the AR have been studied in context of SBMA (Pluciennik et al., 2021) (Figure 1; Table 1). Specifically, ubiquitin-specific peptidase 7 (USP7, a deubiquitinase) has been shown to physically interact with polyQ-AR *in vivo* and *in vitro* (Pluciennik et al., 2021). USP7 overexpression resulted in reduced ubiquitination and enhanced AR aggregation *in vitro* (Pluciennik et al., 2021). In contrast, reducing the expression of USP7 was found to significantly reduce the polyQ-AR aggregation and severity of disease phenotypes in cell, fly, and mouse models of SBMA (Pluciennik et al., 2021). Additionally, the AR K17R mutation was found to enhance the polyQ-AR aggregation and ligand dependent AR stabilization *in vitro* (Pluciennik et al., 2021), which suggested the role of ubiquitination at K17 in degradation and clearance of polyQ-AR. Taken together, these studies identify a critical role for ubiquitination in SBMA pathophysiology.

### SUMOylation

SUMOylation is a process where small ubiquitin-like modifiers (SUMOs, shares close similarity to the ubiquitin proteins) bind to the target proteins and play crucial roles in multiple cellular functions, including neuronal development and differentiation (Henley et al., 2014), DNA damage responses (Jackson and Durocher, 2013), protein quality control (Tatham et al., 2011), and gene expression (Muller et al., 2004) etc. SUMOylation is also known to play critical role in multiple neurodegenerative diseases (Krumova et al., 2011; Vijayakumaran and Pountney, 2018; Qin et al., 2019), including polyQ diseases (Ueda et al., 2002; Steffan et al., 2004; Janer et al., 2010). In the context of SBMA, a study was conducted using fly model to investigate the role of SUMO-1-protein regulatory pathway in modulating the polyQ-AR mediated toxicity (Chan et al., 2002). Expression of a specific mutant form of Ubiquitin-Like Modifier-*Activating Enzyme* 2 (Uba2), a SUMO-1 activating enzyme, was found to worsen the polyQ-AR mediated degeneration in SBMA fly model (Chan et al., 2002); however, it still remains unclear whether the effects of this Uba2 mutant (Uba2.C175S) on SBMA phenotypes are mediated by modulating AR SUMOylation. An another study conducted *in vitro* showed that an increase in SUMOylation significantly reduced mutant AR aggregates without altering the expression levels of AR and AR transcriptional activity (Mukherjee et al., 2009), indicating protective effects of AR SUMOylation in SBMA. In contrast, a later study showed that prevention of SUMOylation in AR was found to be beneficial to SBMA phenotypes *in vivo* (Chua et al., 2015). In this study, a mutant AR knock-in mouse model was made by replacing the conserved lysine sites of SUMOylation with arginine without affecting the ligand dependent nuclear translocation of AR or formation of intranuclear inclusions (Chua et al., 2015). In these animals, potentially prevention of SUMOylation in polyQ-AR showed to enhance the transcriptional activity of AR, rescue type I muscle fiber atrophy, and increase survival (Chua et al., 2015). Taken together, evidence from the previous studies have shown both the beneficial and toxic roles of SUMOylation in SBMA pathogenesis, and thus the exact role of AR-SUMOylation still remains unclear and further investigations are required.

## Therapeutic Perspectives

**FIGURE 2:**
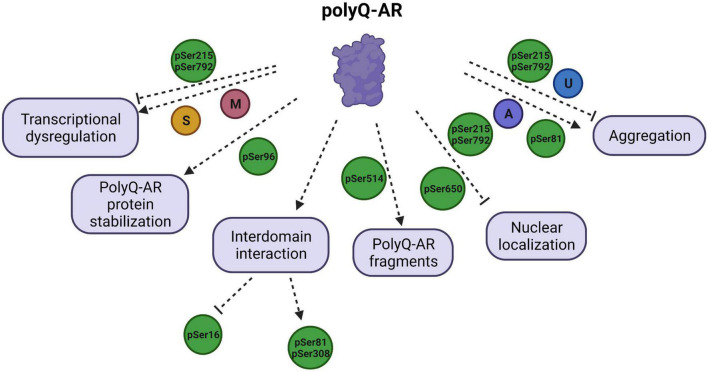
Schematic illustration of polyglutamine (polyQ) -AR functions targeted by PTMs and their effects on the SBMA disease pathology. PTMs regulate the diverse functions of polyQ-AR, and thereby play a critical role in SBMA pathogenesis. Transcriptional dysregulation by polyQ-AR is improved by phosphorylation at S215 (pSer215) and S792 (pSer792) but enhanced by methylation (M) at arginine residues of polyQ-AR by PMRT6. SUMOylation (S) was reported to decrease transactivation of polyQ-AR. Stabilization of polyQ-AR protein is increased by phosphorylation at S96 (pSer96). Phosphorylation of AR at S16 is found to be increased by inhibition of interdomain interaction. AR phosphorylation at S81 and S308 is dependent upon AR N/C interaction. Phosphorylation at S514 (pSer514) leads to formation of toxic polyQ-AR fragments. Nuclear localization of polyQ-AR is reduced by pSer215 and pSer792. Phosphorylation at S650 (pSer650) enhances the nuclear export of polyQ-AR. AR aggregation can be reduced by pSer215, pSer792, ubiquitination (U), but promoted by pSer81 and acetylation (A).

Currently, there are no known cures for SBMA. Available treatment strategies provide only limited improvement, underscoring the need for development of more effective therapeutic options for the patients. There have been several approaches used to modify SBMA pathogenesis, including lowering the levels of androgens such as DHT (Fernandez-Rhodes et al., 2011), modulating the AF2 domain (Badders et al., 2018), targeting the nuclear translocation of polyQ-AR (Giorgetti et al., 2015), inhibiting the oligomerization of polyQ proteins prior to their formation of insoluble aggregates (Minakawa et al., 2020), and various others. Among these multiple strategies is the targeting of PTMs that can affect AR function in various ways and modulate the SBMA disease pathogenesis (Figure 2; Tables 1, 2). The therapeutics targeting the AR function through PTMs to treat SBMA are discussed in this review (Table 2).

The role of phosphorylation as PTM in modulating the polyQ-AR mediated toxicity has shown great potential. A study reported that CDK2 phosphorylates polyQ-AR at S96 site (Polanco et al., 2016). This CDK2 dependent phosphorylation resulted in polyQ-AR protein stabilization and toxicity in a mechanism negatively regulated by AC/PKA signaling pathway (Polanco et al., 2016). In order to apply these findings into therapeutics, an analog of PACAP, which is an activator of AC/PKA pathway, was developed (Polanco et al., 2016). Administration of this analog was able to inhibit the polyQ-AR phosphorylation and decrease polyQ-AR mediated disease toxicity *in vitro* and *in vivo* (Polanco et al., 2016). Similarly, forskolin, a plant-derived activator of AC/PKA pathway, also showed potential in modulating the SBMA disease pathology (Polanco et al., 2016). In *in vitro* models of SBMA, treatment of forskolin reduced phosphorylation of polyQ-AR at S96 and decreased polyQ-AR aggregation (Polanco et al., 2016). Taken together, these findings suggested that the reduction of phosphorylation at S96 in polyQ-AR by activating AC/PKA signaling pathway provides protection against SBMA, indicating polyQ-AR S96 as an important therapeutic target in SBMA.

Previous studies have clearly shown the toxic effect of polyQ-AR in skeletal muscles in SBMA (Yu et al., 2006). Thus, the role of IGF-1, known as a protector of muscle integrity, has also been investigated in SBMA research. In *in vitro* studies, IGF-1 was found to enhance clearance of AR aggregates through the ubiquitin-proteasome system in a mechanism mediated by phosphorylation of AR by Akt (Palazzolo et al., 2009). Overexpression of a muscle-specific IGF-1 isoform was reported to ameliorate behavioral and pathological phenotypes *in vivo* (Palazzolo et al., 2009). Similarly, another study found that administration of mecasermin rinfabate (rhIGF-1/IGFBP3) to SBMA mouse models activated Akt and significantly attenuated the polyQ-AR associated toxicity (Rinaldi et al., 2012). Taking into consideration the beneficial effects of IGF-1 in SBMA pathogenesis, further attempts were made to translate these findings into translational research. The protective effect of IGF-1 in SBMA through BVS857 (a drug, phosphomimetic of IGF-1) was tested in the clinical trials. Unfortunately, no major changes were observed in terms of muscle strength or muscle function in the patients (Grunseich et al., 2018).

The results obtained from the clinical trials further raised questions on whether targeting the PTMs can be utilized in potential therapeutics to treat SBMA. The safety and efficiency of therapeutics utilizing this mechanism and their performance in the clinical trials are still major concerns. Thus, it is important that the therapeutic candidates are extensively tested and validated for their therapeutic potential in *in vivo* and *in vitro* models. An in-depth understanding of the exact roles of PTMs on AR in SBMA context may help in developing more efficient and reliable therapeutics for the SBMA patients in the future.

## Conclusion

Since the genetic underpinnings of SBMA were identified over 30 years ago, our understanding of the molecular and cellular mechanisms underlying SBMA pathogenesis has advanced substantially. Multiple mechanisms have been implicated in SBMA pathogenesis, including structural alterations in AR, transcriptional dysregulation, altered protein-protein interactions, and formation of toxic polyQ-AR fragments and AR aggregation. PTMs of AR have been of particular research interest, as dysfunction of PTM regulation is implicated in AR dysfunction and subsequent SBMA pathology, and manipulation of specific AR PTM sites have shown potential in modulating SBMA pathogenesis as effective therapeutic strategies. Although our understanding of diverse PTM sites of AR, as well as the functional impact of manipulating these sites, has advanced substantially, the translation of these findings to viable therapeutic options for SBMA patients has achieved limited success only. Absence of effective methods to detect the novel and precise PTM sites may serve as a major limitation factor in this aspect. The advancements in genomic and proteomic techniques may help in identification and use of more accurate methods that may facilitate detection of novel and accurate PTM sites that are difficult to access and have yet remained to be discovered. Besides the PTMs discussed in this review, the roles of additional PTMs in AR biology and SBMA disease are the potential areas that may provide more insights in disease pathogenesis and thus needs to be explored. It is also imperative to understand the effect of interactions of PTMs at protein level in regulating AR function to understand the complexity of the disease better. Furthermore, the development of *in vivo* and *in vitro* models that accurately model all aspects of the disease may also aid in more effective translation of findings to therapeutic options for the patients. Gaining detailed insights in these aspects may help discern the gaps in terms of regulatory mechanisms that are involved in SBMA pathogenesis and may aid in the detection of novel biomarkers, drug candidates, or effective and reliable therapeutics to treat SBMA.

## Author Contributions

NG and JL developed the concept. NG, LN, and VO were involved in writing, reviewing, and editing the manuscript. FH and KL were involved in preparation of figures, tables, and editing the manuscript. JL was involved in reviewing, editing, and providing comments on the manuscript. All authors contributed to the article and approved the submitted version.

## Conflict of Interest

The authors declare that the research was conducted in the absence of any commercial or financial relationships that could be construed as a potential conflict of interest.

## Publisher’s Note

All claims expressed in this article are solely those of the authors and do not necessarily represent those of their affiliated organizations, or those of the publisher, the editors and the reviewers. Any product that may be evaluated in this article, or claim that may be made by its manufacturer, is not guaranteed or endorsed by the publisher.

## References

[B1] Abel A. Walcott J. Woods J. Duda J. Merry D. E. (2001). Expression of expanded repeat androgen receptor produces neurologic disease in transgenic mice. *Hum. Mol. Genet.* 10 107–116. 10.1093/hmg/10.2.107 11152658

[B2] Adachi H. Kume A. Li M. Nakagomi Y. Niwa H. Do J. (2001). Transgenic mice with an expanded CAG repeat controlled by the human AR promoter show polyglutamine nuclear inclusions and neuronal dysfunction without neuronal cell death. *Hum. Mol. Genet.* 10 1039–1048. 10.1093/hmg/10.10.1039 11331614

[B3] Anbalagan M. Huderson B. Murphy L. Rowan B. G. (2012). Post-translational modifications of nuclear receptors and human disease. *Nucl. Recept. Signal.* 10:e001. 10.1621/nrs.10001 22438791 PMC3309075

[B4] Arakhamia T. Lee C. E. Carlomagno Y. Duong D. M. Kundinger S. R. Wang K. (2020). Posttranslational modifications mediate the structural diversity of tauopathy strains. *Cell* <*refvol*> 180:e612. 10.1016/j.cell.2020.01.027 32032505 PMC7491959

[B5] Ardito F. Giuliani M. Perrone D. Troiano G. Lo Muzio L. (2017). The crucial role of protein phosphorylation in cell signaling and its use as targeted therapy (Review). *Int. J. Mol. Med.* 40 271–280. 10.3892/ijmm.2017.3036 28656226 PMC5500920

[B6] Arnold F. J. Pluciennik A. Merry D. E. (2019). Impaired nuclear export of polyglutamine-expanded androgen receptor in spinal and bulbar muscular atrophy. *Sci. Rep.* 9:119. 10.1038/s41598-018-36784-4 30644418 PMC6333819

[B7] Atsuta N. Watanabe H. Ito M. Banno H. Suzuki K. Katsuno M. (2006). Natural history of spinal and bulbar muscular atrophy (SBMA): a study of 223 Japanese patients. *Brain* 129(Pt 6) 1446–1455. 10.1093/brain/awl096 16621916

[B8] Badders N. M. Korff A. Miranda H. C. Vuppala P. K. Smith R. B. Winborn B. J. (2018). Selective modulation of the androgen receptor AF2 domain rescues degeneration in spinal bulbar muscular atrophy. *Nat. Med.* 24 427–437. 10.1038/nm.4500 29505030 PMC5975249

[B9] Basso M. Pennuto M. (2015). Serine phosphorylation and arginine methylation at the crossroads to neurodegeneration. *Exp. Neurol.* 271 77–83. 10.1016/j.expneurol.2015.05.003 25979114

[B10] Bedford M. T. Clarke S. G. (2009). Protein arginine methylation in mammals: who, what, and why. *Mol. Cell* 33 1–13. 10.1016/j.molcel.2008.12.013 19150423 PMC3372459

[B11] Beitel L. K. Alvarado C. Mokhtar S. Paliouras M. Trifiro M. (2013). Mechanisms mediating spinal and bulbar muscular atrophy: investigations into polyglutamine-expanded androgen receptor function and dysfunction. *Front. Neurol.* 4:53. 10.3389/fneur.2013.00053 23720649 PMC3654311

[B12] Bevan C. L. Hughes I. A. Patterson M. N. (1997). Wide variation in androgen receptor dysfunction in complete androgen insensitivity syndrome. *J. Steroid Biochem. Mol. Biol.* 61 19–26. 10.1016/s0960-0760(97)00001-0 9328206

[B13] Bielecki B. Mattern C. Ghoumari A. M. Javaid S. Smietanka K. Abi Ghanem C. (2016). Unexpected central role of the androgen receptor in the spontaneous regeneration of myelin. *Proc. Natl. Acad. Sci. U.S.A.* 113 14829–14834. 10.1073/pnas.1614826113 27930320 PMC5187716

[B14] Bingham P. M. Scott M. O. Wang S. McPhaul M. J. Wilson E. M. Garbern J. Y. (1995). Stability of an expanded trinucleotide repeat in the androgen receptor gene in transgenic mice. *Nat. Genet.* 9 191–196. 10.1038/ng0295-191 7719348

[B15] Blanc R. S. Richard S. (2017). Arginine methylation: the coming of age. *Mol. Cell* 65 8–24. 10.1016/j.molcel.2016.11.003 28061334

[B16] Bott L. C. Badders N. M. Chen K. L. Harmison G. G. Bautista E. Shih C. C. (2016). A small-molecule Nrf1 and Nrf2 activator mitigates polyglutamine toxicity in spinal and bulbar muscular atrophy. *Hum. Mol. Genet.* 25 1979–1989. 10.1093/hmg/ddw073 26962150 PMC5062587

[B17] Brooks B. P. Paulson H. L. Merry D. E. Salazar-Grueso E. F. Brinkmann A. O. Wilson E. M. (1997). Characterization of an expanded glutamine repeat androgen receptor in a neuronal cell culture system. *Neurobiol. Dis.* 3 313–323. 10.1006/nbdi.1997.0126 9173928

[B18] Brown C. J. Goss S. J. Lubahn D. B. Joseph D. R. Wilson E. M. French F. S. (1989). Androgen receptor locus on the human X chromosome: regional localization to Xq11-12 and description of a DNA polymorphism. *Am. J. Hum. Genet.* 44 264–269. 2563196 PMC1715398

[B19] Cary G. A. La Spada A. R. (2008). Androgen receptor function in motor neuron survival and degeneration. *Phys. Med. Rehabil. Clin. N. Am.* 19 479–494. 10.1016/j.pmr.2008.03.002 18625411

[B20] Chan H. Y. Warrick J. M. Andriola I. Merry D. Bonini N. M. (2002). Genetic modulation of polyglutamine toxicity by protein conjugation pathways in *Drosophila*. *Hum. Mol. Genet.* 11 2895–2904. 10.1093/hmg/11.23.2895 12393801

[B21] Chang C. Lee S. O. Wang R. S. Yeh S. Chang T. M. (2013). Androgen receptor (AR) physiological roles in male and female reproductive systems: lessons learned from AR-knockout mice lacking AR in selective cells. *Biol. Reprod.* 89:21. 10.1095/biolreprod.113.109132 23782840 PMC4076350

[B22] Chen L. Feany M. B. (2005). Alpha-synuclein phosphorylation controls neurotoxicity and inclusion formation in a *Drosophila* model of Parkinson disease. *Nat. Neurosci.* 8 657–663. 10.1038/nn1443 15834418

[B23] Chen S. Kesler C. T. Paschal B. M. Balk S. P. (2009). Androgen receptor phosphorylation and activity are regulated by an association with protein phosphatase 1. *J. Biol. Chem.* 284 25576–25584. 10.1074/jbc.M109.043133 19622840 PMC2757959

[B24] Chevalier-Larsen E. S. O’Brien C. J. Wang H. Jenkins S. C. Holder L. Lieberman A. P. (2004). Castration restores function and neurofilament alterations of aged symptomatic males in a transgenic mouse model of spinal and bulbar muscular atrophy. *J. Neurosci.* 24 4778–4786. 10.1523/JNEUROSCI.0808-04.2004 15152038 PMC6729468

[B25] Chua J. P. Reddy S. L. Yu Z. Giorgetti E. Montie H. L. Mukherjee S. (2015). Disrupting SUMOylation enhances transcriptional function and ameliorates polyglutamine androgen receptor-mediated disease. *J. Clin. Invest.* 125 831–845. 10.1172/JCI73214 25607844 PMC4319414

[B26] Cohen P. (2002). The origins of protein phosphorylation. *Nat. Cell Biol.* 4 E127–E130. 10.1038/ncb0502-e127 11988757

[B27] Cortes C. J. La Spada A. R. (2018). X-Linked spinal and bulbar muscular atrophy: from clinical genetic features and molecular pathology to mechanisms underlying disease toxicity. *Adv. Exp. Med. Biol.* 1049 103–133. 10.1007/978-3-319-71779-1_5 29427100

[B28] Cortes C. J. Ling S. C. Guo L. T. Hung G. Tsunemi T. Ly L. (2014a). Muscle expression of mutant androgen receptor accounts for systemic and motor neuron disease phenotypes in spinal and bulbar muscular atrophy. *Neuron* 82 295–307. 10.1016/j.neuron.2014.03.001 24742458 PMC4096235

[B29] Cortes C. J. Miranda H. C. Frankowski H. Batlevi Y. Young J. E. Le A. (2014b). Polyglutamine-expanded androgen receptor interferes with TFEB to elicit autophagy defects in SBMA. *Nat. Neurosci.* 17 1180–1189. 10.1038/nn.3787 25108912 PMC4180729

[B30] Davies P. Watt K. Kelly S. M. Clark C. Price N. C. McEwan I. J. (2008). Consequences of poly-glutamine repeat length for the conformation and folding of the androgen receptor amino-terminal domain. *J. Mol. Endocrinol.* 41 301–314. 10.1677/JME-08-0042 18762554

[B31] Deng X. Shao G. Zhang H. T. Li C. Zhang D. Cheng L. (2017). Protein arginine methyltransferase 5 functions as an epigenetic activator of the androgen receptor to promote prostate cancer cell growth. *Oncogene* 36 1223–1231. 10.1038/onc.2016.287 27546619 PMC5322258

[B32] Dossena M. Bedini G. Rusmini P. Giorgetti E. Canazza A. Tosetti V. (2014). Human adipose-derived mesenchymal stem cells as a new model of spinal and bulbar muscular atrophy. *PLoS One* 9:e112746. 10.1371/journal.pone.0112746 25392924 PMC4231043

[B33] Drazic A. Myklebust L. M. Ree R. Arnesen T. (2016). The world of protein acetylation. *Biochim. Biophys. Acta* 1864 1372–1401. 10.1016/j.bbapap.2016.06.007 27296530

[B34] El Kharraz S. Dubois V. van Royen M. E. Houtsmuller A. B. Pavlova E. Atanassova N. (2021). The androgen receptor depends on ligand-binding domain dimerization for transcriptional activation. *EMBO Rep.* 22:e52764. 10.15252/embr.202152764 34661369 PMC8647150

[B35] Feng Y. Maity R. Whitelegge J. P. Hadjikyriacou A. Li Z. Zurita-Lopez C. (2013). Mammalian protein arginine methyltransferase 7 (PRMT7) specifically targets RXR sites in lysine- and arginine-rich regions. *J. Biol. Chem.* 288 37010–37025. 10.1074/jbc.M113.525345 24247247 PMC3873558

[B36] Fernandez-Rhodes L. E. Kokkinis A. D. White M. J. Watts C. A. Auh S. Jeffries N. O. (2011). Efficacy and safety of dutasteride in patients with spinal and bulbar muscular atrophy: a randomised placebo-controlled trial. *Lancet Neurol.* 10 140–147. 10.1016/S1474-4422(10)70321-5 21216197 PMC3056353

[B37] Fu M. Liu M. Sauve A. A. Jiao X. Zhang X. Wu X. (2006). Hormonal control of androgen receptor function through SIRT1. *Mol. Cell Biol.* 26 8122–8135. 10.1128/MCB.00289-06 16923962 PMC1636736

[B38] Funderburk S. F. Shatkina L. Mink S. Weis Q. Weg-Remers S. Cato A. C. (2009). Specific N-terminal mutations in the human androgen receptor induce cytotoxicity. *Neurobiol. Aging* 30 1851–1864. 10.1016/j.neurobiolaging.2007.12.023 18289734

[B39] Gaughan L. Stockley J. Wang N. McCracken S. R. Treumann A. Armstrong K. (2011). Regulation of the androgen receptor by SET9-mediated methylation. *Nucleic Acids Res.* 39 1266–1279. 10.1093/nar/gkq861 20959290 PMC3045589

[B40] Gioeli D. Paschal B. M. (2012). Post-translational modification of the androgen receptor. *Mol. Cell Endocrinol.* 352 70–78. 10.1016/j.mce.2011.07.004 21820033

[B41] Gioeli D. Black B. E. Gordon V. Spencer A. Kesler C. T. Eblen S. T. (2006). Stress kinase signaling regulates androgen receptor phosphorylation, transcription, and localization. *Mol. Endocrinol.* 20 503–515. 10.1210/me.2005-0351 16282370

[B42] Giorgetti E. Rusmini P. Crippa V. Cristofani R. Boncoraglio A. Cicardi M. E. (2015). Synergic prodegradative activity of Bicalutamide and trehalose on the mutant androgen receptor responsible for spinal and bulbar muscular atrophy. *Hum. Mol. Genet.* 24 64–75. 10.1093/hmg/ddu419 25122660 PMC4262493

[B43] Gottlieb L. Guo L. Shorter J. Marmorstein R. (2021). N-alpha-acetylation of Huntingtin protein increases its propensity to aggregate. *J. Biol. Chem.* 297:101363. 10.1016/j.jbc.2021.101363 34732320 PMC8640455

[B44] Grunseich C. Miller R. Swan T. Glass D. J. El Mouelhi M. Fornaro M. (2018). Safety, tolerability, and preliminary efficacy of an IGF-1 mimetic in patients with spinal and bulbar muscular atrophy: a randomised, placebo-controlled trial. *Lancet Neurol.* 17 1043–1052. 10.1016/S1474-4422(18)30320-X 30337273 PMC6415539

[B45] Grunseich C. Zukosky K. Kats I. R. Ghosh L. Harmison G. G. Bott L. C. (2014). Stem cell-derived motor neurons from spinal and bulbar muscular atrophy patients. *Neurobiol. Dis.* 70 12–20. 10.1016/j.nbd.2014.05.038 24925468 PMC4172362

[B46] Gupta R. Sahu M. Srivastava D. Tiwari S. Ambasta R. K. Kumar P. (2021). Post-translational modifications: regulators of neurodegenerative proteinopathies. *Ageing Res. Rev.* 68:101336. 10.1016/j.arr.2021.101336 33775891

[B47] Haigis M. C. Sinclair D. A. (2010). Mammalian sirtuins: biological insights and disease relevance. *Annu. Rev. Pathol.* 5 253–295. 10.1146/annurev.pathol.4.110807.092250 20078221 PMC2866163

[B48] Harada N. Takagi T. Nakano Y. Yamaji R. Inui H. (2015). Protein arginine methyltransferase 10 is required for androgen-dependent proliferation of LNCaP prostate cancer cells. *Biosci. Biotechnol. Biochem.* 79 1430–1437. 10.1080/09168451.2015.1025035 25799006

[B49] Henley J. M. Craig T. J. Wilkinson K. A. (2014). Neuronal SUMOylation: mechanisms, physiology, and roles in neuronal dysfunction. *Physiol. Rev.* 94 1249–1285. 10.1152/physrev.00008.2014 25287864 PMC4187031

[B50] Huang H. Tindall D. J. (2002). The role of the androgen receptor in prostate cancer. *Crit. Rev. Eukaryot. Gene Expr.* 12 193–207. 10.1615/critreveukaryotgeneexpr.v12.i3.30 12449343

[B51] Igarashi S. Tanno Y. Onodera O. Yamazaki M. Sato S. Ishikawa A. (1992). Strong correlation between the number of CAG repeats in androgen receptor genes and the clinical onset of features of spinal and bulbar muscular atrophy. *Neurology* 42 2300–2302. 10.1212/wnl.42.12.2300 1461383

[B52] Jackson S. P. Durocher D. (2013). Regulation of DNA damage responses by ubiquitin and SUMO. *Mol. Cell* 49 795–807. 10.1016/j.molcel.2013.01.017 23416108

[B53] Janer A. Werner A. Takahashi-Fujigasaki J. Daret A. Fujigasaki H. Takada K. (2010). SUMOylation attenuates the aggregation propensity and cellular toxicity of the polyglutamine expanded ataxin-7. *Hum. Mol. Genet.* 19 181–195. 10.1093/hmg/ddp478 19843541

[B54] Jenster G. van der Korput H. A. Trapman J. Brinkmann A. O. (1995). Identification of two transcription activation units in the N-terminal domain of the human androgen receptor. *J. Biol. Chem.* 270 7341–7346. 10.1074/jbc.270.13.7341 7706276

[B55] Jesko H. Wencel P. Strosznajder R. P. Strosznajder J. B. (2017). Sirtuins and their roles in brain aging and neurodegenerative disorders. *Neurochem. Res.* 42 876–890. 10.1007/s11064-016-2110-y 27882448 PMC5357501

[B56] Jochum T. Ritz M. E. Schuster C. Funderburk S. F. Jehle K. Schmitz K. (2012). Toxic and non-toxic aggregates from the SBMA and normal forms of androgen receptor have distinct oligomeric structures. *Biochim. Biophys. Acta* 1822 1070–1078. 10.1016/j.bbadis.2012.02.006 22366762

[B57] Ju H. Kokubu H. Lim J. (2014). Beyond the glutamine expansion: influence of posttranslational modifications of ataxin-1 in the pathogenesis of spinocerebellar ataxia type 1. *Mol. Neurobiol.* 50 866–874. 10.1007/s12035-014-8703-z 24752589 PMC4821199

[B58] Ju H. Kokubu H. Todd T. W. Kahle J. J. Kim S. Richman R. (2013). Polyglutamine disease toxicity is regulated by Nemo-like kinase in spinocerebellar ataxia type 1. *J. Neurosci.* 33 9328–9336. 10.1523/JNEUROSCI.3465-12.2013 23719801 PMC3710458

[B59] Kang A. R. Park S. H. Lee S. Choi D. Y. Kim K. P. Song H. K. (2015). A key lysine residue in the AXH domain of ataxin-1 is essential for its ubiquitylation. *Biochim. Biophys. Acta* 1854 356–364. 10.1016/j.bbapap.2015.01.012 25641559

[B60] Karve T. M. Cheema A. K. (2011). Small changes huge impact: the role of protein posttranslational modifications in cellular homeostasis and disease. *J. Amino Acids* 2011:207691. 10.4061/2011/207691 22312457 PMC3268018

[B61] Katsuno M. Adachi H. Kume A. Li M. Nakagomi Y. Niwa H. (2002). Testosterone reduction prevents phenotypic expression in a transgenic mouse model of spinal and bulbar muscular atrophy. *Neuron* 35 843–854. 10.1016/s0896-6273(02)00834-6 12372280

[B62] Katsuno M. Tanaka F. Adachi H. Banno H. Suzuki K. Watanabe H. (2012). Pathogenesis and therapy of spinal and bulbar muscular atrophy (SBMA). *Prog. Neurobiol.* 99 246–256. 10.1016/j.pneurobio.2012.05.007 22609045

[B63] Kennedy W. R. Alter M. Sung J. H. (1968). Progressive proximal spinal and bulbar muscular atrophy of late onset. A sex-linked recessive trait. *Neurology* 18 671–680. 10.1212/wnl.18.7.671 4233749

[B64] Koh S. S. Chen D. Lee Y. H. Stallcup M. R. (2001). Synergistic enhancement of nuclear receptor function by p160 coactivators and two coactivators with protein methyltransferase activities. *J. Biol. Chem.* 276 1089–1098. 10.1074/jbc.M004228200 11050077

[B65] Koide R. Ikeuchi T. Onodera O. Tanaka H. Igarashi S. Endo K. (1994). Unstable expansion of CAG repeat in hereditary dentatorubral-pallidoluysian atrophy (DRPLA). *Nat. Genet.* 6 9–13. 10.1038/ng0194-9 8136840

[B66] Krumova P. Meulmeester E. Garrido M. Tirard M. Hsiao H. H. Bossis G. (2011). Sumoylation inhibits alpha-synuclein aggregation and toxicity. *J. Cell Biol.* 194 49–60. 10.1083/jcb.201010117 21746851 PMC3135405

[B67] Kuil C. W. Berrevoets C. A. Mulder E. (1995). Ligand-induced conformational alterations of the androgen receptor analyzed by limited trypsinization. Studies on the mechanism of antiandrogen action. *J. Biol. Chem.* 270 27569–27576. 10.1074/jbc.270.46.27569 7499218

[B68] La Spada A. (1993). “Spinal and bulbar muscular atrophy,” in *GeneReviews((R))*, eds Adam M. P. Ardinger H. H. Pagon R. A. Wallace S. E. Bean L. J. H. Gripp K. W. (Seattle, WA: University of Washington). 20301508

[B69] La Spada A. R. Peterson K. R. Meadows S. A. McClain M. E. Jeng G. Chmelar R. S. (1998). Androgen receptor YAC transgenic mice carrying CAG 45 alleles show trinucleotide repeat instability. *Hum. Mol. Genet.* 7 959–967. 10.1093/hmg/7.6.959 9580659

[B70] La Spada A. R. Wilson E. M. Lubahn D. B. Harding A. E. Fischbeck K. H. (1991). Androgen receptor gene mutations in X-linked spinal and bulbar muscular atrophy. *Nature* 352 77–79. 10.1038/352077a0 2062380

[B71] LaFevre-Bernt M. A. Ellerby L. M. (2003). Kennedy’s disease. Phosphorylation of the polyglutamine-expanded form of androgen receptor regulates its cleavage by caspase-3 and enhances cell death. *J. Biol. Chem.* 278 34918–34924. 10.1074/jbc.M302841200 12824190

[B72] Lai J. J. Lai K. P. Zeng W. Chuang K. H. Altuwaijri S. Chang C. (2012). Androgen receptor influences on body defense system *via* modulation of innate and adaptive immune systems: lessons from conditional AR knockout mice. *Am. J. Pathol.* 181 1504–1512. 10.1016/j.ajpath.2012.07.008 22959669 PMC3483803

[B73] Li M. Chevalier-Larsen E. S. Merry D. E. Diamond M. I. (2007). Soluble androgen receptor oligomers underlie pathology in a mouse model of spinobulbar muscular atrophy. *J. Biol. Chem.* 282 3157–3164. 10.1074/jbc.M609972200 17121819

[B74] Lieberman A. P. Harmison G. Strand A. D. Olson J. M. Fischbeck K. H. (2002). Altered transcriptional regulation in cells expressing the expanded polyglutamine androgen receptor. *Hum. Mol. Genet.* 11 1967–1976. 10.1093/hmg/11.17.1967 12165558

[B75] Lieberman A. P. Shakkottai V. G. Albin R. L. (2019). Polyglutamine repeats in neurodegenerative diseases. *Annu. Rev. Pathol.* 14 1–27. 10.1146/annurev-pathmechdis-012418-012857 30089230 PMC6387631

[B76] Lin H. K. Yeh S. Kang H. Y. Chang C. (2001). Akt suppresses androgen-induced apoptosis by phosphorylating and inhibiting androgen receptor. *Proc. Natl. Acad. Sci. U.S.A.* 98 7200–7205. 10.1073/pnas.121173298 11404460 PMC34646

[B77] Lorenzetti D. Bohlega S. Zoghbi H. Y. (1997). The expansion of the CAG repeat in ataxin-2 is a frequent cause of autosomal dominant spinocerebellar ataxia. *Neurology* 49 1009–1013. 10.1212/wnl.49.4.1009 9339681

[B78] Macdonald M. E. Ambrose C. M. Duyao M. P. Myers R. H. Lin C. Srinidhi L. (1993). A novel gene containing a trinucleotide repeat that is expanded and unstable on huntingtons-disease chromosomes. *Cell* 72 971–983. 10.1016/0092-8674(93)90585-E 8458085

[B79] Malena A. Pennuto M. Tezze C. Querin G. D’Ascenzo C. Silani V. (2013). Androgen-dependent impairment of myogenesis in spinal and bulbar muscular atrophy. *Acta Neuropathol.* 126 109–121. 10.1007/s00401-013-1122-9 23644820

[B80] Martin D. D. O. Kay C. Collins J. A. Nguyen Y. T. Slama R. A. Hayden M. R. (2018). A human huntingtin SNP alters post-translational modification and pathogenic proteolysis of the protein causing Huntington disease. *Sci. Rep.* 8:8096. 10.1038/s41598-018-25903-w 29802276 PMC5970160

[B81] Matsumoto T. Sakari M. Okada M. Yokoyama A. Takahashi S. Kouzmenko A. (2013). The androgen receptor in health and disease. *Annu. Rev. Physiol.* 75 201–224. 10.1146/annurev-physiol-030212-183656 23157556

[B82] Matsumoto T. Takeyama K. Sato T. Kato S. (2005). Study of androgen receptor functions by genetic models. *J. Biochem.* 138 105–110. 10.1093/jb/mvi118 16091584

[B83] McManamny P. Chy H. S. Finkelstein D. I. Craythorn R. G. Crack P. J. Kola I. (2002). A mouse model of spinal and bulbar muscular atrophy. *Hum. Mol. Genet.* 11 2103–2111. 10.1093/hmg/11.18.2103 12189162

[B84] Merry D. E. Kobayashi Y. Bailey C. K. Taye A. A. Fischbeck K. H. (1998). Cleavage, aggregation and toxicity of the expanded androgen receptor in spinal and bulbar muscular atrophy. *Hum. Mol. Genet.* 7 693–701. 10.1093/hmg/7.4.693 9499423

[B85] Meyer R. Wolf S. S. Obendorf M. (2007). PRMT2, a member of the protein arginine methyltransferase family, is a coactivator of the androgen receptor. *J. Steroid Biochem. Mol. Biol.* 107 1–14. 10.1016/j.jsbmb.2007.05.006 17587566

[B86] Mhatre A. N. Trifiro M. A. Kaufman M. Kazemi-Esfarjani P. Figlewicz D. Rouleau G. (1993). Reduced transcriptional regulatory competence of the androgen receptor in X-linked spinal and bulbar muscular atrophy. *Nat. Genet.* 5 184–188. 10.1038/ng1093-184 8252045

[B87] Migazzi A. Scaramuzzino C. Anderson E. N. Tripathy D. Hernandez I. H. Grant R. A. (2021). Huntingtin-mediated axonal transport requires arginine methylation by PRMT6. *Cell Rep.* 35:108980. 10.1016/j.celrep.2021.108980 33852844 PMC8132453

[B88] Milioto C. Malena A. Maino E. Polanco M. J. Marchioretti C. Borgia D. (2017). Beta-agonist stimulation ameliorates the phenotype of spinal and bulbar muscular atrophy mice and patient-derived myotubes. *Sci. Rep.* 7:41046. 10.1038/srep41046 28117338 PMC5259768

[B89] Minakawa E. N. Popiel H. A. Tada M. Takahashi T. Yamane H. Saitoh Y. (2020). Arginine is a disease modifier for polyQ disease models that stabilizes polyQ protein conformation. *Brain* 143 1811–1825. 10.1093/brain/awaa115 32436573

[B90] Monks D. A. Johansen J. A. Mo K. Rao P. Eagleson B. Yu Z. (2007). Overexpression of wild-type androgen receptor in muscle recapitulates polyglutamine disease. *Proc. Natl. Acad. Sci. U.S.A.* 104 18259–18264. 10.1073/pnas.0705501104 17984063 PMC2084330

[B91] Montie H. L. Pestell R. G. Merry D. E. (2011). SIRT1 modulates aggregation and toxicity through deacetylation of the androgen receptor in cell models of SBMA. *J. Neurosci.* 31 17425–17436. 10.1523/JNEUROSCI.3958-11.2011 22131404 PMC6088793

[B92] Mookerjee S. Papanikolaou T. Guyenet S. J. Sampath V. Lin A. Vitelli C. (2009). Posttranslational modification of ataxin-7 at lysine 257 prevents autophagy-mediated turnover of an N-terminal caspase-7 cleavage fragment. *J. Neurosci.* 29 15134–15144. 10.1523/JNEUROSCI.4720-09.2009 19955365 PMC2907146

[B93] Mukherjee S. Thomas M. Dadgar N. Lieberman A. P. Iniguez-Lluhi J. A. (2009). Small ubiquitin-like modifier (SUMO) modification of the androgen receptor attenuates polyglutamine-mediated aggregation. *J. Biol. Chem.* 284 21296–21306. 10.1074/jbc.M109.011494 19497852 PMC2755854

[B94] Muller S. Ledl A. Schmidt D. (2004). SUMO: a regulator of gene expression and genome integrity. *Oncogene* 23 1998–2008. 10.1038/sj.onc.1207415 15021887

[B95] Nakamura K. Jeong S. Y. Uchihara T. Anno M. Nagashima K. Nagashima T. (2001). SCA17, a novel autosomal dominant cerebellar ataxia caused by an expanded polyglutamine in TATA-binding protein. *Hum. Mol. Genet.* 10 1441–1448. 10.1093/hmg/10.14.1441 11448935

[B96] Nath S. R. Yu Z. Gipson T. A. Marsh G. B. Yoshidome E. Robins D. M. (2018). Androgen receptor polyglutamine expansion drives age-dependent quality control defects and muscle dysfunction. *J. Clin. Invest.* 128 3630–3641. 10.1172/JCI99042 29809168 PMC6063498

[B97] Nedelsky N. B. Pennuto M. Smith R. B. Palazzolo I. Moore J. Nie Z. (2010). Native functions of the androgen receptor are essential to pathogenesis in a Drosophila model of spinobulbar muscular atrophy. *Neuron* 67 936–952. 10.1016/j.neuron.2010.08.034 20869592 PMC3514079

[B98] Orr C. R. Montie H. L. Liu Y. Bolzoni E. Jenkins S. C. Wilson E. M. (2010). An interdomain interaction of the androgen receptor is required for its aggregation and toxicity in spinal and bulbar muscular atrophy. *J. Biol. Chem.* 285 35567–35577. 10.1074/jbc.M110.146845 20826791 PMC2975181

[B99] Orr H. T. Zoghbi H. Y. (2007). Trinucleotide repeat disorders. *Annu. Rev. Neurosci.* 30 575–621. 10.1146/annurev.neuro.29.051605.113042 17417937

[B100] Orr H. T. Chung M. Y. Banfi S. Kwiatkowski T. J. Jr. Servadio A. Beaudet A. L. (1993). Expansion of an unstable trinucleotide CAG repeat in spinocerebellar ataxia type 1. *Nat. Genet.* 4 221–226. 10.1038/ng0793-221 8358429

[B101] Palazzolo I. Burnett B. G. Young J. E. Brenne P. L. La Spada A. R. Fischbeck K. H. (2007). Akt blocks ligand binding and protects against expanded polyglutamine androgen receptor toxicity. *Hum. Mol. Genet.* 16 1593–1603. 10.1093/hmg/ddm109 17470458

[B102] Palazzolo I. Nedelsky N. B. Askew C. E. Harmison G. G. Kasantsev A. G. Taylor J. P. (2010). B2 attenuates polyglutamine-expanded androgen receptor toxicity in cell and fly models of spinal and bulbar muscular atrophy. *J. Neurosci. Res.* 88 2207–2216. 10.1002/jnr.22389 20336775 PMC3881232

[B103] Palazzolo I. Stack C. Kong L. Musaro A. Adachi H. Katsuno M. (2009). Overexpression of IGF-1 in muscle attenuates disease in a mouse model of spinal and bulbar muscular atrophy. *Neuron* 63 316–328. 10.1016/j.neuron.2009.07.019 19679072 PMC2735765

[B104] Pennuto M. Basso M. (2016). In vitro and in vivo modeling of spinal and bulbar muscular atrophy. *J. Mol. Neurosci.* 58 365–373. 10.1007/s12031-015-0677-4 26614347

[B105] Pennuto M. Palazzolo I. Poletti A. (2009). Post-translational modifications of expanded polyglutamine proteins: impact on neurotoxicity. *Hum. Mol. Genet.* 18 R40–R47. 10.1093/hmg/ddn412 19297400

[B106] Pluciennik A. Liu Y. Molotsky E. Marsh G. B. Ranxhi B. Arnold F. J. (2021). Deubiquitinase USP7 contributes to the pathogenicity of spinal and bulbar muscular atrophy. *J. Clin. Invest.* 131:e134565. 10.1172/JCI134565 33170804 PMC7773404

[B107] Polanco M. J. Parodi S. Piol D. Stack C. Chivet M. Contestabile A. (2016). Adenylyl cyclase activating polypeptide reduces phosphorylation and toxicity of the polyglutamine-expanded androgen receptor in spinobulbar muscular atrophy. *Sci. Transl. Med.* 8:370ra181. 10.1126/scitranslmed.aaf9526 28003546 PMC11349029

[B108] Qin M. Li H. Bao J. Xia Y. Ke D. Wang Q. (2019). SET SUMOylation promotes its cytoplasmic retention and induces tau pathology and cognitive impairments. *Acta Neuropathol. Commun.* 7:21. 10.1186/s40478-019-0663-0 30767764 PMC6376727

[B109] Ramazi S. Zahiri J. (2021). Posttranslational modifications in proteins: resources, tools and prediction methods. *Database* 2021:baab012. 10.1093/database/baab012 33826699 PMC8040245

[B110] Ramzan F. McPhail M. Rao P. Mo K. Halievski K. Swift-Gallant A. (2015). Distinct etiological roles for myocytes and motor neurons in a mouse model of kennedy’s disease/spinobulbar muscular atrophy. *J. Neurosci.* 35 6444–6451. 10.1523/JNEUROSCI.3599-14.2015 25904795 PMC6605215

[B111] Ratovitski T. Jiang M. O’Meally R. N. Rauniyar P. Chighladze E. Farago A. (2021). Interaction of huntingtin (HTT) with PRMTs and its subsequent arginine methylation affects HTT solubility, phase transition behavior and neuronal toxicity. *Hum. Mol. Genet.* 31, 1651–1672. 10.1093/hmg/ddab351 34888656 PMC9122652

[B112] Rinaldi C. Bott L. C. Chen K. L. Harmison G. G. Katsuno M. Sobue G. (2012). Insulinlike growth factor (IGF)-1 administration ameliorates disease manifestations in a mouse model of spinal and bulbar muscular atrophy. *Mol. Med.* 18 1261–1268. 10.2119/molmed.2012.00271 22952056 PMC3521783

[B113] Saporita A. J. Zhang Q. Navai N. Dincer Z. Hahn J. Cai X. (2003). Identification and characterization of a ligand-regulated nuclear export signal in androgen receptor. *J. Biol. Chem.* 278 41998–42005. 10.1074/jbc.M302460200 12923188

[B114] Scaramuzzino C. Casci I. Parodi S. Lievens P. M. J. Polanco M. J. Milioto C. (2015). Protein arginine methyltransferase 6 enhances polyglutamine-expanded androgen receptor function and toxicity in spinal and bulbar muscular atrophy. *Neuron* 85 88–100. 10.1016/j.neuron.2014.12.031 25569348 PMC4305189

[B115] Shaffer P. L. Jivan A. Dollins D. E. Claessens F. Gewirth D. T. (2004). Structural basis of androgen receptor binding to selective androgen response elements. *Proc. Natl. Acad. Sci. U.S.A.* 101 4758–4763. 10.1073/pnas.0401123101 15037741 PMC387321

[B116] Shang Y. Myers M. Brown M. (2002). Formation of the androgen receptor transcription complex. *Mol. Cell* 9 601–610. 10.1016/s1097-2765(02)00471-9 11931767

[B117] Simeoni S. Mancini M. A. Stenoien D. L. Marcelli M. Weigel N. L. Zanisi M. (2000). Motoneuronal cell death is not correlated with aggregate formation of androgen receptors containing an elongated polyglutamine tract. *Hum. Mol. Genet.* 9 133–144. 10.1093/hmg/9.1.133 10587588

[B118] Sopher B. L. Thomas P. S. Jr. LaFevre-Bernt M. A. Holm I. E. Wilke S. A. Ware C. B. (2004). Androgen receptor YAC transgenic mice recapitulate SBMA motor neuronopathy and implicate VEGF164 in the motor neuron degeneration. *Neuron* 41 687–699. 10.1016/s0896-6273(04)00082-0 15003169

[B119] Soraru G. D’Ascenzo C. Polo A. Palmieri A. Baggio L. Vergani L. (2008). Spinal and bulbar muscular atrophy: skeletal muscle pathology in male patients and heterozygous females. *J. Neurol. Sci.* 264 100–105. 10.1016/j.jns.2007.08.012 17854832

[B120] Steffan J. S. Agrawal N. Pallos J. Rockabrand E. Trotman L. C. Slepko N. (2004). SUMO modification of Huntingtin and Huntington’s disease pathology. *Science* 304 100–104. 10.1126/science.1092194 15064418

[B121] Stenoien D. L. Cummings C. J. Adams H. P. Mancini M. G. Patel K. DeMartino G. N. (1999). Polyglutamine-expanded androgen receptors form aggregates that sequester heat shock proteins, proteasome components and SRC-1, and are suppressed by the HDJ-2 chaperone. *Hum. Mol. Genet.* 8 731–741. 10.1093/hmg/8.5.731 10196362

[B122] Sternburg E. L. Gruijs da Silva L. A. Dormann D. (2022). Post-translational modifications on RNA-binding proteins: accelerators, brakes, or passengers in neurodegeneration? *Trends Biochem. Sci.* 47 6–22. 10.1016/j.tibs.2021.07.004 34366183

[B123] Sun L. Fang J. (2016). Macromolecular crowding effect is critical for maintaining SIRT1’s nuclear localization in cancer cells. *Cell Cycle* 15 2647–2655. 10.1080/15384101.2016.1211214 27463693 PMC5053580

[B124] Suzuki E. Zhao Y. Ito S. Sawatsubashi S. Murata T. Furutani T. (2009). Aberrant E2F activation by polyglutamine expansion of androgen receptor in SBMA neurotoxicity. *Proc. Natl. Acad. Sci. U.S.A.* 106 3818–3822. 10.1073/pnas.0809819106 19237573 PMC2656163

[B125] Suzuki K. Katsuno M. Banno H. Takeuchi Y. Atsuta N. Ito M. (2008). CAG repeat size correlates to electrophysiological motor and sensory phenotypes in SBMA. *Brain* 131(Pt 1) 229–239. 10.1093/brain/awm289 18056738

[B126] Swatek K. N. Komander D. (2016). Ubiquitin modifications. *Cell Res.* 26 399–422. 10.1038/cr.2016.39 27012465 PMC4822133

[B127] Takeyama K. Ito S. Yamamoto A. Tanimoto H. Furutani T. Kanuka H. (2002). Androgen-dependent neurodegeneration by polyglutamine-expanded human androgen receptor in *Drosophila*. *Neuron* 35 855–864. 10.1016/s0896-6273(02)00875-9 12372281

[B128] Tang S. Sethunath V. Metaferia N. Y. Nogueira M. F. Gallant D. S. Garner E. R. (2022). A genome-scale CRISPR screen reveals PRMT1 as a critical regulator of androgen receptor signaling in prostate cancer. *Cell Rep.* 38:110417. 10.1016/j.celrep.2022.110417 35196489 PMC9036938

[B129] Tatham M. H. Matic I. Mann M. Hay R. T. (2011). Comparative proteomic analysis identifies a role for SUMO in protein quality control. *Sci. Signal.* 4:rs4. 10.1126/scisignal.2001484 21693764

[B130] Thomas M. Dadgar N. Aphale A. Harrell J. M. Kunkel R. Pratt W. B. (2004). Androgen receptor acetylation site mutations cause trafficking defects, misfolding, and aggregation similar to expanded glutamine tracts. *J. Biol. Chem.* 279 8389–8395. 10.1074/jbc.M311761200 14670946

[B131] Todd T. W. Kokubu H. Miranda H. C. Cortes C. J. La Spada A. R. Lim J. (2015). Nemo-like kinase is a novel regulator of spinal and bulbar muscular atrophy. *Elife* 4:e08493. 10.7554/eLife.08493 26308581 PMC4577982

[B132] Tsai M. J. O’Malley B. W. (1994). Molecular mechanisms of action of steroid/thyroid receptor superfamily members. *Annu. Rev. Biochem.* 63 451–486. 10.1146/annurev.bi.63.070194.002315 7979245

[B133] Ueda H. Goto J. Hashida H. Lin X. Oyanagi K. Kawano H. (2002). Enhanced SUMOylation in polyglutamine diseases. *Biochem. Biophys. Res. Commun.* 293 307–313. 10.1016/S0006-291X(02)00211-5 12054600

[B134] Vijayakumaran S. Pountney D. L. (2018). SUMOylation, aging and autophagy in neurodegeneration. *Neurotoxicology* 66 53–57. 10.1016/j.neuro.2018.02.015 29490232

[B135] Vinueza-Gavilanes R. Inigo-Marco I. Larrea L. Lasa M. Carte B. Santamaria E. (2020). N-terminal acetylation mutants affect alpha-synuclein stability, protein levels and neuronal toxicity. *Neurobiol. Dis.* 137:104781. 10.1016/j.nbd.2020.104781 31991248

[B136] Walcott J. L. Merry D. E. (2002). Ligand promotes intranuclear inclusions in a novel cell model of spinal and bulbar muscular atrophy. *J. Biol. Chem.* 277 50855–50859. 10.1074/jbc.M209466200 12388541

[B137] Wilkinson K. D. (1987). Protein ubiquitination: a regulatory post-translational modification. *Anticancer Drug Des.* 2 211–229. 2835061

[B138] Wyce A. Bai Y. Nagpal S. Thompson C. C. (2010). Research resource: the androgen receptor modulates expression of genes with critical roles in muscle development and function. *Mol. Endocrinol.* 24 1665–1674. 10.1210/me.2010-0138 20610535 PMC5417449

[B139] Yazawa I. (2000). Aberrant phosphorylation of dentatorubral-pallidoluysian atrophy (DRPLA) protein complex in brain tissue. *Biochem. J.* 351(Pt 3) 587–593. 11042112 PMC1221397

[B140] Yu Z. Dadgar N. Albertelli M. Gruis K. Jordan C. Robins D. M. (2006). Androgen-dependent pathology demonstrates myopathic contribution to the Kennedy disease phenotype in a mouse knock-in model. *J. Clin. Invest.* 116 2663–2672. 10.1172/JCI28773 16981011 PMC1564432

[B141] Zboray L. Pluciennik A. Curtis D. Liu Y. Berman-Booty L. D. Orr C. (2015). Preventing the androgen receptor n/c interaction delays disease onset in a mouse model of SBMA. *Cell Rep.* 13 2312–2323. 26673324 10.1016/j.celrep.2015.11.019 PMC4684905

[B142] Zhou Z. X. Sar M. Simental J. A. Lane M. V. Wilson E. M. (1994). A ligand-dependent bipartite nuclear targeting signal in the human androgen receptor. Requirement for the DNA-binding domain and modulation by NH2-terminal and carboxyl-terminal sequences. *J. Biol. Chem.* 269 13115–13123. 8175737

